# Regulatory T-lymphocyte subsets in children with chronic immune thrombocytopenia after high-dose of dexamethasone

**DOI:** 10.1038/s41390-022-01978-0

**Published:** 2022-02-16

**Authors:** Khalid Ibrahim Elsayh, Khaled Saad, Naglaa Samy Osman, Khaled Hashim Mahmoud, Faisal A. Ahmad, Shaimaa M. Khalaf, Noha G. Sayed, Zeinab Albadry M. Zahran, Aliaa M. A. Ghandour, Amira A. Elhoufey, Tamer Bedir, Asmaa Zahran

**Affiliations:** 1 Department of Pediatrics, Faculty of Medicine, Assiut University, Assiut, Egypt; 2 Department of clinical pathology, Faculty of Medicine, Assiut University, Assiut, Egypt; 3 Department of Medical Microbiology and Immunology, Faculty of Medicine, Assiut University, Assiut, Egypt; 4 Department of Community Health Nursing, Faculty of Nursing, Assiut University, Assiut, Egypt; 5 Department of Community Health Nursing, Alddrab University College, Jazan University, Jazan, Saudi Arabia; 6 Department of Medical Microbiology and Immunology, Faculty of Medicine, Mansoura University, Mansoura, Egypt; 7 Department of Clinical Pathology, South Egypt Cancer Institute, Assiut University, Assiut, Egypt

## Abstract

**Background:**

Immune thrombocytopenia (ITP) is an acquired autoimmune disease. This study’s objective was to estimate the variations in the population of CD4^+^CD25^+High^ FoxP3^+^ cells (CD4^+^ regulatory T-lymphocytes; Tregs) in previously untreated children with chronic ITP managed in Assiut University Hospitals, as well as to evaluate the efficacy of high-dose dexamethasone (HD-DXM) in these patients.

**Methods:**

In this study, we investigated the frequencies of T-lymphocyte subsets in 27 untreated children with chronic ITP.

**Results:**

Prior to treatment, the percentages of CD4^+^CD25^High^ cells and Tregs were significantly lower in the chronic ITP group compared to the control group (*p* = 0.018 and *p* < 0.0001, respectively). After treatment with HD-DXM, Tregs and platelets were significantly increased in these patients (*p* < 0.0001 for both).

**Conclusions:**

Our results suggest that Tregs are deficient in children with chronic ITP and that HD-DXM immunosuppressive therapy can restore the levels of these cells.

**Impact:**

- CD4^+^CD25^High^ cells and Tregs were significantly lower in children chronic ITP compared to healthy control.
- HD-DXM treatment led to significantly increased Tregs and platelets in these patients.
- Our results suggest that Tregs are deficient in children with chronic ITP and that HD-DXM immunosuppressive therapy can restore the levels of these cells.

## Introduction

Pediatric immune thrombocytopenia (ITP) is defined as primary thrombocytopenia (platelets <100 × 10^9^ cell/L) that is found to be caused by an immunological disorder that targets and destroys platelets through several mechanisms after exclusion of secondary causes of thrombocytopenia.^1,2^ ITP is usually an acute disease and has an incidence of 4.2/100,000 children, most of whom are 2–5 years old.^3^ ITP typically has a self-limiting course, but if persists beyond 12 months, it is considered a chronic condition.^4^ Chronic disease is usually observed in 20% of the pediatric population presenting with ITP.^5^ When the disorder is chronic, the main goal is to improve the patients’ quality of life rather than to simply increase their platelet count.^6^

Second lines of treatment for chronic ITP that are used if the traditional first-line treatment fails can include steroid, intravenous immunoglobulin, or a combination of both and splenectomy.^4,7^ Immunosuppressive medications are also a good choice in many patients. Such medications include as rituximab, mycophenolate mofetil, sirolimus, and romiplostim, and eltrombopag.^8–11^ These medications have shown a high response rate in many trials,^4,12^ but their cost and unavailability in low-income countries make the simple and available drugs the best option for low-income countries. Long-term use of steroids is a treatment option, but there are many concerns about their side effects, which include impaired blood glucose levels, increased blood pressure, impaired growth, and osteoporosis.^4^ Due to the affordability and availability of dexamethasone, it can be used for the treatment of chronic ITP in developing countries.

ITP is usually caused by antiplatelet antibodies produced by autoreactive B-lymphocytes that are stimulated and assisted by T-helper lymphocytes (CD4^+^ cells).^13^ Current investigations show that patients with ITP have an imbalance in cytokine levels and antiplatelet self-reactive T-lymphocytes, which indicates a loss of peripheral tolerance.^14^ CD4^+^CD25^+High^ FoxP3^+^ cells (CD4^+^ regulatory T-cells; Tregs) suppress the proliferation of B and T-lymphocytes, thus preventing their unwarranted stimulation and autoreactivity, and insufficiency of Tregs is implicated in peripheral tolerance malfunction, which can result in autoimmunity development.^11^ A deficit in Tregs production and/or function, which can be linked to an intrinsic Tregs defect, was discovered in ITP. Such defects may result in both the failure of Treg-mediated immunological suppression and T-cell responses to platelet autoantigen.^13–15^

As most of the previous clinical trials and researches were in adults and/or acute ITP, the objectives of our study were to assess the variations in the population of Tregs in untreated children with chronic ITP managed in Assiut University Hospitals and to evaluate the effect of high-dose dexamethasone (HD-DXM) on T-cell subsets in these patients.

## Patients and methods

### Study design

Our study was a prospective interventional clinical trial that was carried out in Assiut University Children’s Hospital. Twenty-seven pediatric patients known to have chronic ITP were recruited from December 2020 to the end of May 2021. They were diagnosed as having chronic ITP, as their platelet counts were <100 × 10^9^ cell/L for more than one year, with no other hematological abnormalities or organomegaly,^6^ and secondary causes of ITP such as infections, pediatric immunodeficiency disorders, connective tissue diseases such as systemic lupus erythematous, malignancies, drug-induced thrombocytopenia, and congenital thrombocytopenia were excluded.^4,6^

### High-dose dexamethasone treatment protocol

Eligible patients received intravenous dexamethasone (24 mg/m^2^ in a single dose) every two weeks for six cycles after admission to the Hematology Unit. This regimen was recommended from our unit with experience of previous work.^16^ The patients were followed up during the treatment, with measurement of the vital signs (pulse, blood pressure), blood glucose, and electrolytes, as well as assessment for any skin changes or psychic disturbances, to detect any complications. The outcome, platelet count, was measured at the end of the course of treatment. A platelet count of ≥100 × 10^9^/L with no clinically relevant bleeding was defined as remission or complete response to treatment.^17^ No steroid or immunosuppressive treatments had been given to any of the patients prior to the study.

Twenty-one healthy subjects of comparable age and sex distribution served as a control group, and all of the patients and controls received a comprehensive history, focusing on previous medications, bleeding symptoms and signs, and ITP grading;^14^ in addition, complete physical examinations were carried out to exclude any signs of possible alternative diagnoses.

Routine laboratory assessments, including CBC with a blood film, liver and kidney function, urine analysis, and serology for HBV and HCV, were performed. In addition, flow cytometric analysis of regulatory T-lymphocyte subsets was performed before and after treatment with HD-DXM.

The study was conducted as per the Declaration of Helsinki, and the Ethics Committee of the Faculty of Medicine, Assiut University, reviewed and approved the research (No. 17300668-2021). Written consent was obtained from caregivers of the children with chronic ITP and healthy controls. Blood samples were taken from the enrolled patients prior to HD-DXM for flow cytometric analysis of lymphocyte subsets, and another sample was taken three days after completion of the last cycle.

### Flow cytometric detection of regulatory T-cells in peripheral blood

**Fig. 1:**
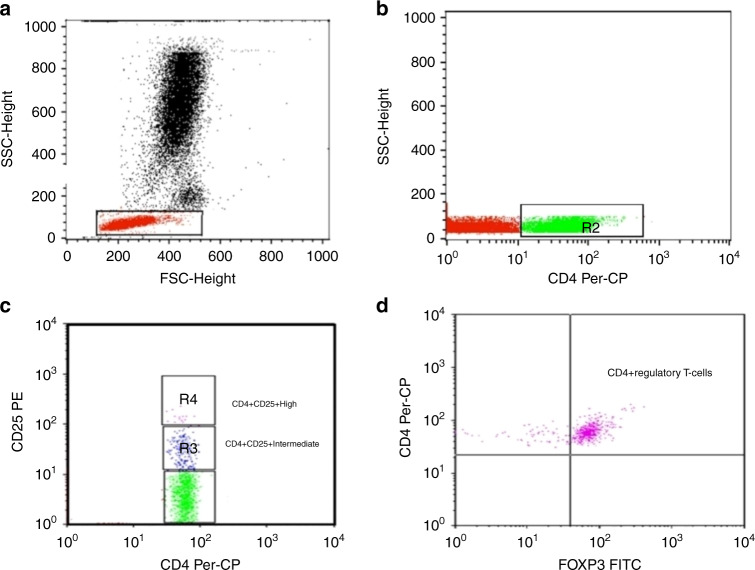
Flow cytometric detection of regulatory T-cells. **a** Scatter histogram was used to define the lymphocytes population (R1). **b** CD4^+^ cells were detected on the lymphocyte population and then gated for further analysis. **c** The expression of CD25 on CD4^+^ then detected, and different gates were drawn to define CD4^+^CD25^+Intermediate^ cells, and CD4^+^CD25^+High^ cells. **d** Then the percentage of CD4^+^CD25^+High^ FoxP3^+^ cells (CD4^+^ regulatory T-cells) was determined.

Fifty µl of blood was stained with 5 µL of phycoerythrin (PE)-conjugated CD25 (IQ Product, Netherlands) and peridinium-chlorophyll-protein (Per-CP)-conjugated-CD4 (Becton Dickinson [BD] Biosciences, CA). After 20 min of incubation at 4 °C in the dark, red blood cells were lysed, and the remaining cells were washed with phosphate-buffered saline (PBS). Fixation solution was then added to the tube, and the cells were incubated for 10 minutes. The cells were then washed, and 5 µL of fluoroisothiocyanate (FITC)-conjugated Foxp3 (BD, Bioscience, CA) was added to the same tube, which was incubated for 20 min. Next, the cells were resuspended in PBS and analyzed using a FACSCalibur flow cytometer with CellQuest software (BD Biosciences, CA). An isotype-matched IgG-negative control was used for each sample. Tregs were calculated as the percentage of CD4^+^ cells, as illustrated in Fig. 1.

### Statistical analysis

Data are expressed as mean ± standard deviation (SD) or median (range). Paired independent sample *t*-tests were used for the comparisons. Tests in which the *p*-value was <0.05 indicated a significant difference.

## Results

Table 1 shows the baseline demographic, clinical, and hematological parameters of chronic ITP patients and controls. Our study included 27 children with chronic ITP (14 males; 52%) between the ages of 3 and 13 (mean age 5.65 ± 2.98). Purpuric skin lesions were present in all patients, and mild epistaxis was found in 43%. Platelet count, and hemoglobin were significantly lower in patients than in healthy children, while mean platelet volume (MPV) is significantly higher in patients than control (Table 1). Regarding other hematological parameters, total leukocytic count was comparable between patients and control (Table 1).

**Table 1:** Baseline demographic and hematological parameters of chronic ITP patients and controls.

	Patients (*n* = 27)	Controls (*n* = 21)	*p*-value
Age	5.65 ± 2.98	6.07 ± 3.12	0.64
Sex (male/female)	14/13	11/10	0.31
Petechial rash	27/27	0/21	–
Bruising	27/27	0/21	–
Epistaxis (mild)	11/27	0/21	–
Splenomegaly	0/27	0/21	–
Hepatomegaly	0/27	0/21	–
Platelet count (10^9^/L)	12.09 ± 4.68	278.39 ± 76.54	<0.0001*
MPV (fL)	11.13 ± 1.99	8.43 ± 1.57	<0.0001*
Hemoglobin (g/dL)	10.15 ± 1.15	11.47 ± 1.16	<0.0001*
Total leukocyte count (10^9^/L)	6.88 ± 1.85	7.44 ± 1.96	0.313
CD19 lymphocytes	14.32 ± 1.32	11.62 ± 2.12	0.000*

Data are represented as mean ± SD, independent sample *t*-test was used in comparison.

*n* number, *L* liter, *MPV* mean platelet volume, *fL* femtoliter, *g* gram, *dL* deciliter, *WBCs* white blood cells.

*Significant.

**Fig. 2:**
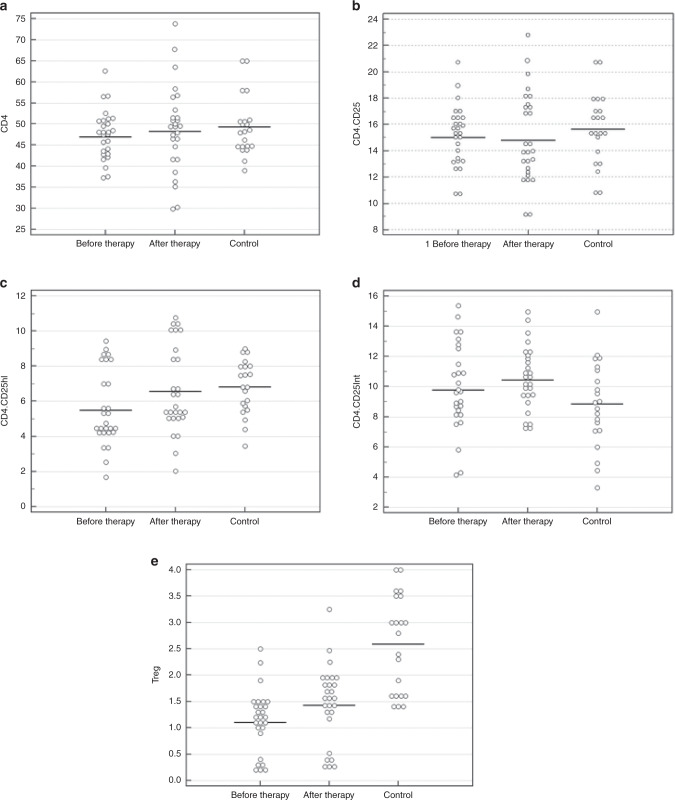
T-lymphocyte’s subsets in chronic ITP patients pre and post-treatment and controls. **a** The percentages of CD4 were comparable between the ITP patients (46.95 ± 5.04) and control (49.32 ± 6.99), also no significant change is observed after HD-DXM in chronic ITP patients (48.16 ± 9.74). **b** The percentages of CD4^+^CD25^+^ were comparable between the ITP patients (15.04 ± 2.21) and control (15.65 ± 2.72), also no significant change is observed after HD-DXM in chronic ITP patients (14.78 ± 3.308). **c** The percentages of CD4^+^CD25^High^ were significantly lower in the ITP patients (5.47 ± 2.07) versus that of control (6.80 ± 1.54), and it significantly increased after HD-DXM in chronic ITP patients (6.57 ± 2.48) with *p*-value < 0.0001. **d** The percentages of CD4^+^CD25^Intermediate^ T-cells were comparable between the ITP patients (9.76 ± 2.79) and control (8.85 ± 2.87), also no significant change is observed after HD-DXM in chronic ITP patients (10.43 ± 1.99). **e** The percentages of regulatory T-cells were significantly lower in the ITP patients (1.10 ± 0.56) versus that of control (2.58 ± 0.93), and it significantly increased after HD-DXM in chronic ITP patients (1.43 ± 0.73) with *p*-value < 0.0001.

The percentages of CD19^+^ B-lymphocytes were significantly higher in patients than controls (Table 1), while the percentages of CD4^+^, CD4^+^CD25^+^, and CD4^+^CD25^Intermediate^ T-cells were comparable between patients and controls (Fig. 2a–c), but the percentages of CD4^+^CD25^High^ cells and Tregs were significantly lower in the chronic ITP group in comparison to control group (Fig. 2). The mean platelet count was significantly increased after therapy (*p* < 0.0001). Twenty-two patients (81.5%) have platelet count ≥150 × 10^9^/L and the rest of the patients have platelet count between 60 and <150 × 10^9^/L.

Figure 2 shows that the percentages of CD4^+^CD25^High^ cells and Tregs were also significantly increased after therapy (*p* < 0.0001 for each) as seen in (Fig. 2). There was no significant difference in the percentage of Tregs (out of all CD4^+^ cells) between the healthy children and the patient group after HD-DXM therapy. However, the percentages of CD4^+^, CD4^+^CD25^+^, and CD4^+^CD25^Intermediate^ T-cells showed no significant changes after treatment (Fig. 2). Overall, our study found that HD-DXM significantly increased the platelet count, CD4^+^CD25^High^ T-cells, and CD4^+^Foxp3^+^ Tregs in children with chronic ITP.

## Discussion

ITP pathogenesis has been thought to be antibody mediated. Several T-cell abnormalities, including polarization toward T1 subsets and changes in the number of Tregs and Th17 cells, have been described in ITP.^18,19^ Our results demonstrated that T-cell subsets in children with chronic ITP were dysregulated. The percentages of CD4^+^CD25^High^ cells and Tregs were significantly lower in the chronic ITP group compared to the control group. After HD-DXM treatment, the percentages of these cells increased significantly. HD-DXM generated a good response in children with chronic ITP in our study, which was consistent with earlier reports.^20–23^ Among the mechanisms that mediate the effect of high-dose steroids on platelets in chronic ITP are their antagonistic effect on macrophage differentiation, suppression of the reticulo-endothelial system’s phagocytic activity, induction of lymphopenia, inhibition of activated T-lymphocytes, and reduction in autoantibody production.^22,23^ Prior to treatment, the number of CD4^+^Foxp3^+^ Tregs was significantly lower in children with chronic ITP. The frequencies of CD4^+^Foxp3^+^ Tregs increased after treatment with HD-DXM. These data suggest that Treg insufficiency may play a role in the pathogenesis of the active phase of chronic ITP, which can be improved by HD-DXM.

There is growing evidence that impairment of Tregs plays a significant role in the pathogenesis of chronic ITP,^24^ however, the previous studies were mainly in adult patients. Our findings support these findings in a relatively large cohort of children with chronic ITP. In adult ITP, many investigations have shown a decreased frequency of Tregs or an imbalance of circulating T-helper cell-associated cytokines, implying the loss of peripheral immunological tolerance. In the last 20 years, studies on the potential relationship between Tregs and platelet counts in both adults and children and in acute and chronic ITP have shown diverse results. Liu et al.^3^ found that adult patients with acute ITP had significantly lower Tregs than patients who had reached remission, with no significant difference between patients in remission and healthy controls. They reported that the suppressive activity of Tregs was impaired in ITP patients. Moreover, ITP patients have a high Th1/Th2 ratio and a Th1 cytokine bias. Another study^1^ reported that Tregs were significantly lower in children with acute and chronic ITP compared to controls. In addition, patients with chronic ITP and platelet counts >100 × 10^9^/L had higher Treg counts than patients with thrombocytopenia (<100 × 10^9^/L), and corticosteroid-responsive patients had higher Treg counts than those whose disease was not responsive. In contrast to our findings and those of previous reports, a few studies^25,26^ found that the difference in the frequency of CD4^+^CD25^High^Foxp3^+^ Tregs was not statistically significant between patients and controls; moreover, there was no difference in Treg counts between acute and chronic groups and no significant correlations between Tregs and platelet counts in the patient and control groups.

Still in line with our results, Li et al.^21^ found that patients with chronic ITP had significantly lower Tregs than healthy controls. In addition, they reported that HD-DXM restored the Th1/Th2 ratio, as well as the number of Th17 and Tregs, to normal levels. Dexamethasone was also shown to adjust T-cell subset levels by suppressing GATA3 and FOXp3 expression while boosting RORt expression.

Understanding the pathophysiology and the mechanisms of HD-DXM treatment in children with chronic ITP can help clinicians make more successful clinical decisions, especially in low-income developing countries. Future studies are needed to address the discrepancies in the results of the studies just discussed and our findings.

## Conclusion

Our results indicated that children with chronic ITP have a defective Treg compartment and that HD-DXM immunosuppressive therapy could restore the levels of these cells.
